# On the Proposed Abolition of Private Asylums

**Published:** 1853-10-01

**Authors:** 


### ON THE PROPOSED ABOLITION OF PRIVATE ASYLUMS.

The following remarks, taken from the last chapter of Dr. Duncan's useful and valuable work, entitled, "*On Popular Errors on the subject of Insanity,*" are so confirmatory of the view taken by ourselves of this question in the first article in our present number, that we with pleasure place them before our readers.

There remains yet one other suggestion to be considered in connexion with this subject, and that is, the propriety of suppressing all the existing private lunatic asylums, and establishing governmental institutions of a corresponding description in their stead, for the accommodation of patients in the higher classes of society.........

I think it may be taken for granted, after what has been already stated, that there exists no such decided necessity for the adoption of further precautions against unjustifiable confinement as to force us to adopt a more stringent system of lunatic asylums, without regard to the consequences to which such system may lead. Matters are well enough provided for already to lead us to pause before embarking in a new, expensive, and complicated machinery, unless it can be shown that it is at least equally advantageous in other respects with that which it is intended to supplant. Now, the proposed plan of having only governmental asylums seems open to this objection, that it would be likely to lead the relatives of the insane to hesitate even more than they do at present before placing their lunatic friends in places where they can be properly treated. When we consider the probable size of such establishments, their public character, their being governed by an official, and numerous board, &c., we can scarcely doubt that such a feeling would be produced, and that a large section of the community would refuse to avail themselves of the advantages they would present, and that sooner than send their friends there, they would endeavour to get them treated in some private place. It is needless to say that such a course would entirely defeat the intention of those who now advocate the formation of such establishments. If a door is now open to abuse and cruelty, the practical effect of the new arrangement would be to open it still more widely, while, as we have formerly seen, it would be decidedly injurious to many patients, by depriving them of the only effective means for having their malady properly treated. Nor could the inconvenience be remedied by any law that could be framed, making it penal to have lunatics kept in private houses. So long as any patients of this class are permitted to be at large, it would be obviously impossible to have its provisions constructed in such a manner as to allow of a distinction being drawn between those who might and those who might not be confined in separate lodgings.

The admission of the principle that such a thing as a proprietary asylum is not to be permitted to exist, plainly involves the hypothesis that the government is prepared to establish different institutions in every requisite variety of style of accommodation, and rate of charge, to suit the different classes of society, and the pecuniary circumstances of individual patients. Admitting that the government were even to do this, the question remains to be asked—Will they create several asylums of each grade, and by doing so, leave the parties requiring accommodation the power of selecting between rival institutions, so as to have in some measure the option of disposing their invalid relative where they may think most for his advantage? or will they, by creating one only of each kind, virtually establish a monopoly which they must necessarily be satisfied with? I say nothing of the difficulty of managing the receipts and expenditure of such establishments, so as to secure to each individual the full amount of the comforts to which his payments entitle him, and at the same time to guard against their becoming an expense to the State; for I take it for granted that it would never be contemplated that the rates of charge should be so arranged as to leave a profit to the country; and if not, how is the calculation to be made, so as at all times, and under the varying conditions of a fluctuating number of inmates, to guard against a deficiency? Difficulties still greater would probably arise, as to the extent of country for which such institutions should be constructed, which would be peculiarly embarrassing if the friends of the patient were left an option of using them or not; and if such an option were not accorded to them, the whole proceedings would have an arbitrary and tyrannical character, altogether foreign from the spirit of the constitution.

There is a plain and obvious reason why the Government should establish public asylums for the use of the poor, because the inability of the classes for whose benefit such establishments are intended, to provide proper accommodation for their reception without such assistance, leaves the country only a choice of evils—either to suffer the neglected lunatics to wander about the country uncared for and uncontrolled, a burden to themselves, and a source of danger to others, or to undertake the duty of providing proper accommodation for them at the public expense. These establishments, consequently, partake essentially of an eleemosynary character. But no such necessity can be urged for the adoption of a similar system of public asylums for the rich; and although they might not, in any proper sense of the word, deserve to be considered as charitable institutions, yet it cannot be forgotten that the sturdy spirit of independence which forms so striking a feature in the national character, would strenuously resist the attempt to force upon them the use of a public and state provision for the accommodation of their relatives and friends; and it seems scarcely possible to prevent many persons taking a wrong idea of the nature of these institutions when they perceive others precisely similar formed on a basis of a purely charitable nature. In these countries there is a strong feeling among persons in independent circumstances against allowing the State any control over what they consider their proper and personal rights; and it has consequently become a settled maxim in the policy of the country, that the Government is not to be asked to do anything that can be done equally well by private enterprise.

It must not be forgotten that if the proprietors of private asylums have a direct pecuniary interest in the success of their establishments, this very circumstance, though it may occasionally betray them into improprieties, is of itself a powerful stimulus to exert themselves to the utmost to promote their efficiency as places of recovery. The surest step to success in this as in every other undertaking, is to prove oneself deserving of it. A large number of cures effected in a short time will do infinitely more to fill the purse of the proprietor than all the questionable gains he can lay his hands on, by detaining patients unnecessarily after they have recovered, or admitting others who ought never to have been received. In a public institution it is obvious no such stimulus can exist. A sense of duty, and a desire of reputation, are the only motives that can prompt a man to exertion, whose salary is fixed, and independent of the issue. These, undoubtedly, are sufficient to induce many who occupy this honourable position to use their utmost ability for the good of the patients committed to their care; yet, looking at human nature generally, we cannot help admitting that the sense of a man's income depending upon his exertions in any undertaking is a motive infinitely more powerful in effect, though lower in moral principle, in leading him to discharge his duties zealously and efficiently. Even the ambition to earn personal reputation has less scope to act in the case of managers of a public institution than in that of the proprietors of a private one; because in the latter the character of the establishment is simply and exclusively that of the individual who conducts it. It is impossible to separate the one from the other. There is no prestige arising from honourable names associated in the management, and identified with its interests, to obscure, by the weight of rank, influence, or authority, the exact measure of personal fitness for the situation he fills. In public asylums, on the contrary, the managing board occupy the prominent place in the public eye; they are the source of authority, and the fixed and permanent head, while the manager is but a subordinate officer, occupying the post for a season, and removable at pleasure. It is hence much more difficult for him to bring his personal exertions into public notice, so as to earn for himself a distinct and honourable character. Beneath the shade of the official directors, both the deficiencies and the merits of the managing superintendent are concealed from general observation, and consequently the stimulus which would otherwise be so valuable in stirring him up to individual effort, loses a great part of its real value.

There is one obvious advantage that private asylums possess over public ones, which deserves to be noticed, and that is, that the entire power of directing their concerns, as well as the sole responsibility, is placed in the hands of a single individual. Hence all the details of their management are characterized by unity of action and design—a matter of the very greatest importance in the treatment of lunatics. If the assistants hesitate or refuse to comply with the views of the head of the institution, there is a short and simple method of getting rid of the inconvenience. This is not always the case in public institutions. Subordinate officers often occasion a great deal of annoyance to the principal superintendent, and thwart his wishes in a variety of ways, which it is difficult to bring under the notice of the board, or to represent to them in such a manner as to convince them of the necessity of adopting an effectual remedy for the evil complained of. This is not the fault of lunatic asylums exclusively, but of all public institutions governed by a numerous committee, the members of which do not always see matters in the same light or act harmoniously together. Parties will sometimes be formed among them, and one official is backed by one party, while another is backed by a different party, and the worst consequences are necessarily produced. It is a fortunate circumstance when the manager so far possesses the confidence of the board under which he is placed, as to find himself uniformly supported in the just exercise of his legitimate authority.

Still further, in the carrying into execution of projected improvements, the proprietor of a private asylum is not hampered by having to consult and to obtain the co-operation of a board who may not feel the importance of what his more practical mind may show him to be necessary for the benefit of the patients. Many circumstances conduce with the directors of public institutions generally, to postpone or refuse the adoption of plans for the improvement of the trust committed to their charge. There is the expenditure of money in the first instance, which may not be easily obtained, the problematical character of the benefit to be secured, the temporary inconvenience that it would occasion, the subsequent expenses it may lead to, and a thousand other little reasons that weigh as an incubus upon the project, and lead them to prefer letting matters remain as they are, at least for a little longer, until perhaps the success of the experiment elsewhere awakens them from their lethargy, or some other circumstance leads them to listen favourably to the proposal. The proprietor of a private asylum, on the contrary, may adopt, on his own responsibility, and at his own impulse, any alteration in the system of management, style of accommodation, and construction of the buildings, that he thinks likely to be conducive to the comfort and health of the patients. There is no delay nor difficulty in carrying it into execution. The only limits to his passion for improvement are those which spring from the length of his purse, and the encouragement he may think the public are disposed to give to such an expenditure. But herein consists the real inferiority of private to public asylums; the former, being merely mercantile speculations, set on foot to answer a present, and, it may be, a passing purpose, do not possess the permanence that is requisite to justify a large outlay of capital in their construction. Being capable of being soon diverted to another purpose, from the death or failure of the proprietor, it would not do to embark a large sum in the erection of buildings, and fitting-out of pleasure-grounds, &c., which could not be usefully applied to any object but that for which it was originally planned. Public asylums, on the other hand, being national undertakings, deliberately resolved on, and not being likely to be diverted from their original design, naturally must command all the advantages that a generous supply of public money can secure.

